# Direct eye gaze enhances the ventriloquism effect

**DOI:** 10.3758/s13414-022-02468-5

**Published:** 2022-03-31

**Authors:** Nadine Lavan, Wing Yue Chan, Yongping Zhuang, Isabelle Mareschal, Sukhwinder S. Shergill

**Affiliations:** 1 Department of Biological and Experimental Psychology, School of Biological and Behavioural Sciences, Queen Mary University of London, Mile End Road, London, E1 4NS UK; 2 Department of Psychosis Studies, Institute of Psychiatry, Psychology & Neuroscience, King’s College London, London, UK; 3 Kent and Medway Medical School, Canterbury, UK

**Keywords:** Ventriloquism effect, Sound localization, Eye gaze, Direct gaze, Voice

## Abstract

The “ventriloquism effect” describes an illusory phenomenon where the perceived location of an auditory stimulus is pulled toward the location of a visual stimulus. Ventriloquists use this phenomenon to create an illusion where an inanimate puppet is perceived to speak. Ventriloquists use the expression and suppression of their own and the puppet’s mouth movements as well the direction of their respective eye gaze to maximize the illusion. While the puppet’s often exaggerated mouth movements have been demonstrated to enhance the ventriloquism effect, the contribution of direct eye gaze remains unknown. In Experiment 1, participants viewed an image of a person’s face while hearing a temporally synchronous recording of a voice originating from different locations on the azimuthal plane. The eyes of the facial stimuli were either looking directly at participants or were closed. Participants were more likely to misperceive the location of a range of voice locations as coming from a central position when the eye gaze of the facial stimuli were directed toward them. Thus, direct gaze enhances the ventriloquist effect by attracting participants’ perception of the voice locations toward the location of the face. In an exploratory analysis, we furthermore found no evidence for an other-race effect between White vs Asian listeners. In Experiment 2, we replicated the effect of direct eye gaze on the ventriloquism effect, also showing that faces per se attract perceived sound locations compared with audio-only sound localization. Showing a modulation of the ventriloquism effect by socially-salient eye gaze information thus adds to previous findings reporting top-down influences on this effect.

## Introduction

Ventriloquism performances capitalize on multisensory integration whereby the location of a visual stimulus can influence the perceived location of an auditory stimulus. As a result, a ventriloquist can create a powerful illusion, where their voice is perceived to originate from an inanimate puppet instead of the actual source. Many studies have replicated this so-called ventriloquism effect in the laboratory by presenting spatially-discrepant audiovisual stimuli to participants and asking them to locate the spatial origin of the auditory stimulus. As is experienced during a ventriloquism performance, these studies reliably report that the perceived location of the auditory stimulus is shifted toward the location of the visual stimulus (for reviews, see Bruns, 2019; Chen & Vroomen, 2013).

The ventriloquism effect, however, does not rely on the stimuli being a (human) voice and face, nor does the auditory and visual stimulus pairings need to be meaningfully linked. The ventriloquism effect can be readily induced using low-level audiovisual stimuli such as bursts of broadband noise or light flashes (e.g., Montagne & Zhou, 2018; Thomas, 1941; Vroomen & De Gelder, 2004). Indeed, even light flashes presented outside of the awareness of participants have been shown to affect the perceived location of sounds (Delong et al., 2018). Since the above studies use pairings of relatively arbitrary or meaningless audiovisual stimuli, they have been taken to suggest that the ventriloquism effect is likely underpinned by low-level sensory integration or cross-modal interactions. Perhaps as a result of the low-level nature of the effect, there have been a number of studies suggesting that the effect is relatively immune to top-down cognitive factors such as attention to the visual stimuli (Bertelson et al., 2000; Vroomen et al., 2001), visual stimulus manipulations, such as face inversions (Bertelson et al., 1994; Colin et al., 2001) or the degree of phonetic (in)congruency between speech sounds and mouth movements (cf. the McGurk effect; Bertelson et al., 1994; McGurk & MacDonald, 1976; but see Kanaya & Yokosawa, 2011).

However, in parallel, there are other studies that do report evidence for top-down influences on the ventriloquism effect—sometimes reporting on similar effects as the studies described above (e.g., Bruns, 2019, for a review). For example, the semantic congruency between the auditory and visual stimuli can enhance the ventriloquism effect. As such, the location of the sound of a dog bark is perceived as being closer to the location of the picture of a dog than to, for example, the picture of a car (Delong & Noppeney, 2021). Note, however, that other, earlier studies report that semantic congruency (a voice paired with a face vs. a voice paired with light flashes) between the audio-visual stimuli does not significantly affect sound localizations in the presence of visual stimuli (Radeau & Bertelson, 1977). The ventriloquism effect is also enhanced by the synchronicity and/or congruency between the production of speech and mouth movements (Driver, 1996; Thurlow & Jack, 1973). Furthermore, linking reward to enhanced performance on a task has been shown to modulate the ventriloquism effect (Bruns et al., 2014). From this brief literature review, it is therefore apparent that the ventriloquism effect can be (1) readily elicited by pairings of auditory and visual stimuli and (2) can under some circumstances be modulated by experimental factors, such as task and stimulus properties.

Classic ventriloquism performances using a puppet often emulate a similar style of interaction between the ventriloquist and the puppet. When “talking,” the inanimate puppet is usually perched on the ventriloquist’s lap, facing the audience while moving its mouth in time with the ventriloquist’s speech. At the same time, the ventriloquist is suppressing their own mouth movements and is looking at the puppet, facing away from the audience. When the ventriloquist speaks as themself, they usually then turn to look at the audience and speak without suppression of mouth movements, while the puppet is presented as looking at the ventriloquist. These features of ventriloquism performances maximize the strength of the illusion by directing the audiences’ attention away from the ventriloquist and toward the puppet. Previous laboratory studies have already shown that the presence of (synchronous) mouth movements can indeed enhance the ventriloquism effect (Driver, 1996; Thurlow & Jack, 1973). Similarly, Borjon et al. (2011) and Vroomen and Stekelenburg (2014) have shown that directed eye gaze can affect perceptual sound localizations, such that a sound’s perceived location can be shifted toward the direction of the eye gaze. Note, however, that this effect is not consistently observed (Vroomen & Stekelenburg, 2014). Independent of whether the effect is observed or not, these studies are in fact not a demonstration of the classic ventriloquism effect per se since the perception of the location of the auditory stimuli is *pushed* away from the visual stimuli and toward the direction of the eye gaze but is, crucially, not *pulled* toward the location of the visual stimuli (i.e., the image of the face). The papers, however, nonetheless demonstrate that the ventriloquist’s gaze being directed at the puppet can aid the illusion. What is still unclear is whether the saliency of the puppet’s “interaction” with the audience as determined by the puppet’s direction of gaze (i.e., facing the audience) also contributes to the strength of the ventriloquism effect.

Many studies have highlighted the importance of direct (mutual) eye gaze as a social and self-relevant cue (Hamilton, 2016). It serves to determine the focus of attention of another person (Friesen et al., 2005), exercise social control (Kleinke, 1986), signals turn taking in conversation (Argyle & Cook, 1976), and aids the inference of mental states of others (Baron-Cohen, 1995). Furthermore, direct gaze can facilitate the rapid orienting of attention toward the face (e.g., Mares et al., 2016). In terms of audio-visual integration, studies have also shown that direct gaze can support speech intelligibility, such that a speaker is more readily understood when they are looking at the listener (Holler et al., 2014; but see McGettigan et al., 2017). Similarly, a recent study (Wahn et al., 2021), has shown that direct gaze enhances the McGurk effect. Thus, participants more frequently reported to have perceived the syllable /da/ in the presence of direct haze, even though they had been, for example, presented with a stimulus that included a voice recording of a person saying /ba/ in the auditory modality and a video of a person saying /ga/.

Given the critical role that direct gaze appears to play during classic ventriloquism performances, during multisensory integration, and more generally in regulating social interactions, we sought to examine whether direct gaze can influence the listeners’ perception of a voice’s location. Specifically, we asked whether the presence of direct gaze would enhance the ventriloquism effect over and above the presence of a face that does not exhibit direct gaze. To do this, we ran two experiments: In Experiment 1, participants were presented with recordings of voices originating from nine spatial locations on the azimuthal plane while viewing the face of a person who was either looking directly at them or had their eyes closed. In Experiment 2, we replicated this experiment, adding an auditory-only baseline condition where no face was present on the screen to contextualise our effect within the broader literature on the ventriloquism effect. In both experiments, participants were asked to indicate the perceived location of the voice. Building on the findings from the gaze perception and sound localization literature, we predicted for both experiments that when the face displayed direct gaze, participants would perceive the voice as coming from a more central location than when the eyes were shut. Thus, direct gaze would pull participants’ perception of the location of the voice toward the location of the face and result in a larger ventriloquism effect. For Experiment 2, we predicted that the perceived location of a voice should be pulled toward the location of the face, be it with eyes closed or exhibiting direct gaze, compared with the audio-only baseline condition, where no visual stimulus was present.

## Experiment 1

For Experiment 1, we additionally posed a secondary research question: In this experiment, half of the participants were White while the other half of the participants were East Asian. All partcipants were presented with stimuli of White faces. This sampling strategy was linked to another task completed by participants in the same testing session (see Methods). We therefore explored whether the participants’ ethnicity (East Asian vs White) would modulate any effects of direct gaze on voice location perception as a secondary research question. Previous studies have reported other-race effects for face perception (Hancock & Rhodes, 2008; Hugenberg et al., 2010; Rhodes et al., 2009). Similarly, the salience of (direct) gaze perception has been shown to be dependent on whether a face matches the participant’s ethnicity: Collova et al. (2017) show that direct gaze is more readily detectable in own-race faces. Furthermore, Pavan et al. (2011) show that how attention is modulated by direct gaze depends on the ethnicity of the face—with differential patterns arising for Black versus White participants in their study. Notably, the reported effect was, however, only present when ethnicity was situationally salient. Based on this research, we predicted that participant ethnicity could interact with our gaze manipulation: If gaze is more readily detectable in own-race faces (Collova et al., 2017), we could expect a diminished or absent ventriloquist effect for East Asian participants. Conversely, however, if ethnicity only affects direct gaze perception when it is situationally salient (Pavan et al., 2011), we would expect no effect of ethnicity on the ventriloquist effect in this experiment.

### Methods

#### Participants

This experiment formed part of a battery of tests completed by 196 participants. The data from 125 participants is presented in the analysis below (see Data Processing for exclusion criteria). In this sample of 125, 68 participants (mean age = 26.01 years, *SD* = 7.26 years; 23 female, two other) self-identified as White living in Western countries and 57 participants self-identified as Asian (mean age = 25.89 years, *SD* = 8.15 years; 40 female). Forty-seven of these participants reported that they were currently living in China, while the 10 remaining participants came from more diverse locations in East Asia. Participants were recruited via Prolific.co (for the White participants) and via social media and personal contacts (for East Asian participants). All participants were fluent in English, had no self-reported hearing difficulties, and had normal or corrected-to-normal vision.

The study was approved by the local ethics committee at Kings College London (ethics number: MRSU-20/21-22882). All participants were reimbursed for their time (£2.50 for approximately 25 minutes of participation).

#### Materials

##### Voices

**Fig. 1:**
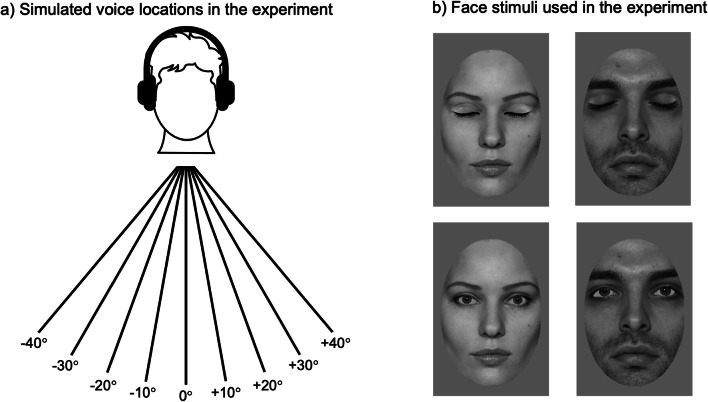
**a** Illustration of the nine different voice locations simulated in the current experiment. **b** A reproduction of the face stimuli that were used in the current experiment. The top row shows the male and female face with their eyes closed; the bottom row shows the two faces looking directly at the camera (DeBruine & Jones, 2017). The auditory and visual stimuli were then paired and presented to participants simultaneously, with the face appearing on a computer screen in front of participants

Participants were presented with a male and a female voice producing the vowel “ah” in a neutral tone of voice. These recordings were selected from the emotionally neutral voice recordings of the Montreal Affective Voices Corpus (Belin et al., 2008). The stimuli had a duration of 221 ms and 195 ms respectively and were root-mean-square (RMS) normalized for intensity using PRAAT (Boersma & Weenink, 2013). The original stimulus recordings were mono recordings, such that we created stereo versions to facilitate further audio manipulations by duplicating the original mono channel. These stereo sounds were then manipulated systematically to simulate a range of different sound locations using the Dear VR micro plugin in REAPER 64. Dear VR is a tool used to, for example, enhance virtual reality experiences as well as making computer games appear more realistic by spatializing the audio. *Dear VR* micro thus enables users to place sounds in 3D space—by, among other processes, changing the relative loudness of the left and right stereo channel to one another. We created 9 versions of each of the 2 voices, with synthesized sound locations ranging from −40° (i.e., the sound was manipulated to be located to the listeners’ left) to +40° (i.e., the sound was manipulated to be located to the listeners’ right) azimuth in 10° steps with 0° elevation (see Fig. 1a). This array of spatial locations was chosen after initial piloting indicated that voice locations of 40° to the left or right of the participant were likely detectable with high accuracy, when presented via a range of headphones. We note, however, that due to, for example, the varied headphones and/or soundcards that participants will have used when completing our experiment online and on their own computers (see Procedure), it is unlikely that the sounds were presented and perceived at exactly the intended locations (−40° to +40° azimuth) across participants. Any reference to the exact sound location in thus paper should, however, be interpreted as an intended sound location, which will not necessarily precisely reflect the perceived sound location. Despite this, our listeners will have nonetheless perceived a graded array of sound locations that varied substantially from left to right.

##### Faces

**Fig. 2:**
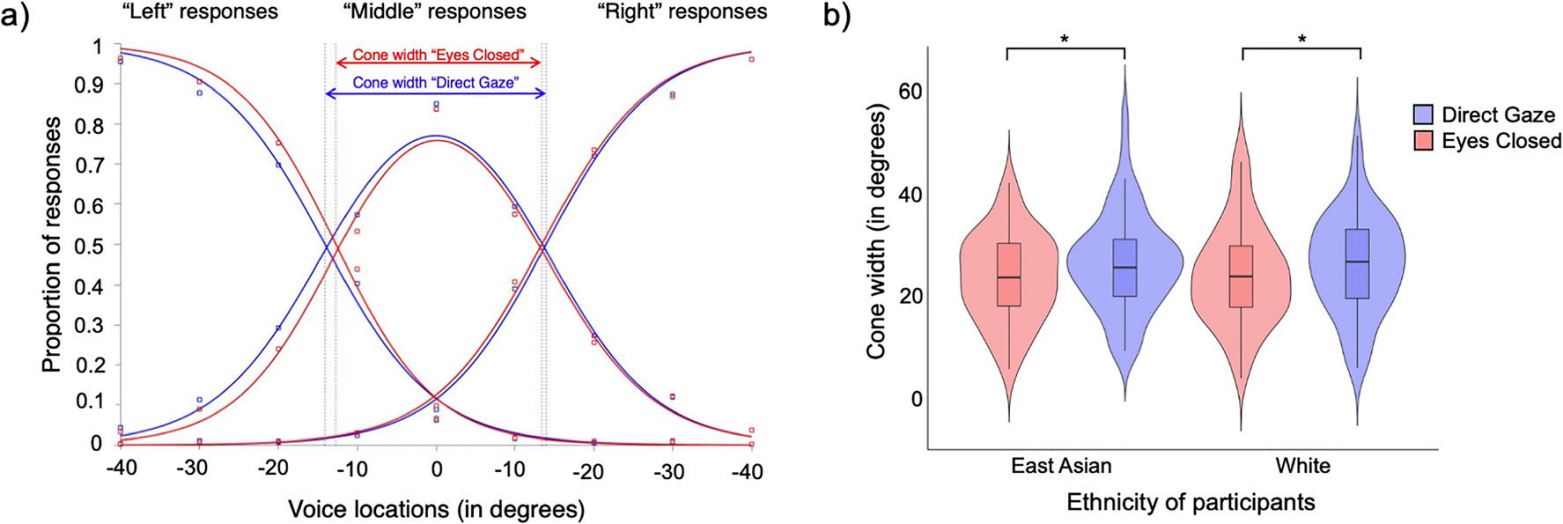
**a** Plot showing the functions fitted for average “left,” “right,” and “middle” responses in the “Eyes Closed” (red) and “Direct Gaze” (blue) conditions. The dashed lines mark the intersections between the functions fitted to the “left” and “right” responses respectively and the functions fitted to the “middle” responses per gaze condition. The arrows show the cone width per gaze condition. **b** Violin plots showing the data by eye gaze condition and participant ethnicity. *p < .05. (Colour figure online)

The voices were presented alongside greyscale images of a male or a female face taken from the Face Research Lab London Set (DeBruine & Jones, 2017). The images were cropped with an oval mask to only show the internal structure of the face. The face stimuli were shown either looking directly at the camera (Direct Gaze condition) or were digitally manipulated in Adobe Photoshop to have their eyes closed (Eyes Closed condition; see Fig. 2b).

#### Procedure

The experiment was implemented using the online experiment builder Gorilla (Anwyl-Irvine et al., 2020), and participants were able to complete the experiment only on a laptop or desktop computer. Participants first read an information sheet and then provided consent to take part in the study. They provided basic demographic information and completed an eye gaze perception task (7 minutes) and two questionnaires (Ames et al., 2006; Mason & Claridge, 2006; 10 minutes). The data from the eye gaze perception task and the questionnaire were part of a separate project and are not analyzed here. Following these tasks, participants were asked to sit directly facing the computer screen and to wear headphones. They were then asked to adjust audio volume of their computer to a comfortable level. To ensure that the binaural sound presentation was working as expected, participants completed a custom-built check, such that, for example, the right-hand phone was indeed placed on the participant’s right ear. This was achieved by presenting participants with sounds played from one phone only (i.e., sounds coming from one side only) and asking them to confirm where the sound was coming from (left or right). Prior to the main task, participants additionally familiarized themselves with the nine simulated sound locations (ranging from −40° to +40° azimuth in 10° steps with 0° degrees of elevation). To achieve this, participants listened to both the male and the female recordings from all simulated spatial locations, while information about the voice location was provided on the screen (e.g., “These two voices are located 30° degrees to your right”). Participants were able to replay the sounds representing each location as many times as they felt necessary. Following this familiarization, participants proceeded to the main task.

In participants completed 180 experimental trials plus 12 vigilance trials (see below). During each trial, they were presented simultaneously with a recording of a voice, originating from one of the nine simulated spatial locations alongside an image of a face that was visible in the centre of the screen for the duration of the voice recording. Each of the nine simulated voice locations was tested 20 times. For each of these 20 trials, participants were presented with a face from one of the two different gaze manipulations, resulting in 10 trials using a face with direct gaze and 10 trials using a face with the eyes closed for that particular location. The face and the voice were gender-matched, and gender was counterbalanced across trials such that half of the trials for each condition included female face–voice pairings and the other half included male face–voice pairings. Using the Gorilla screen calibration tool, the faces were always shown at a width of 6 cm on each participant’s screen.

After the stimulus presentation, participants were asked to determine whether the voice was located to their left, their right, or from a central location in a three-way forced choice paradigm. Responses were self-timed, and participants registered their responses via a key press. After giving a response, a fixation cross appeared for 1,000 ms in the middle of the screen before the task automatically moved on to the next trial.

To monitor participants attention in both the auditory and the visual modality, we included 12 vigilance trails (six auditory, six visual; see exclusions). During auditory vigilance trials, participants were presented with a synthesized voice instructing them to respond to this trial with a specific answer key (e.g., “Please press G”), while a face was also present on the screen. For the visual vigilance trials, participants were shown a text on the screen, also instructing them to respond with a specific key.

#### Data processing

Of the original sample of 196 participants, 71 were excluded: 23 participants failed more than 20% of vigilance trials (see below), indicating that these participants may not have paid sufficient attention to the task; a further 47 participants were removed because accuracy for one or more of the easiest conditions (−40° and +40° azimuth; see Materials) was lower than 80%, suggesting technical issues, poor (spatial) sound delivery, or otherwise low-quality data; and one additional participant was excluded as we were unable to fit required logistic functions to their data (see below). Therefore, our final sample included 125 participants.

The large number of exclusions is perhaps not surprising when collecting data for a task of this nature online (see Methods). The sound localization task requires fine-grained perceptual decisions about auditory locations, which can be affected by a number of factors (e.g., the quality of participants’ headphones and their listening environment), which are difficult to control when doing research online. Importantly, all reported findings hold when using less conservative exclusion criteria (e.g., when including the 47 participants for whom the accuracy was lower than 80% in the easiest conditions in the sample), which suggests that the conservative exclusion criteria are not inherently biasing the reported results.

To examine the effect of eye gaze on voice location perception, we computed the equivalent of the “cone of direct gaze”; the range of gaze deviations a person perceives to be directed toward them (Jun et al., 2013; Mareschal et al., 2013; Stoyanova et al., 2010). For each participant, logistic functions are fitted to the proportion of “left” and “right” responses for the sound data associated with each location in the two conditions separately (direct gaze vs. eyes closed). A function for “middle” responses was calculated by subtracting the proportion of the “left” and “right” responses from one (see Fig. 2a). These three functions were fitted at the same time using the Nelder–Mead simplex method (Nelder & Mead, 1965) implemented in MATLAB using the function *fminsearch*. This method was chosen as it considers the nonindependence of the three functions while also minimizing the residual variance across all three functions. Our measure of interest, the cone width, is defined as the distance (in degrees) between the points where the functions fitted to the proportion of “left” and “right” responses respectively intersect with the “middle” curve (see Fig. 2a). The measure of cone width in our study thus describes the range of voice locations which participants perceived as coming from a central position and overlapping with the position of the face on the screen in front of them. If direct gaze enhances the ventriloquism effect, we would expect the cone width to be wider in the Direct Gaze condition compared with the Eyes Closed condition, with direct eye gaze pulling a wider range of sound locations towards to face.

### Results

To examine the effects of eye gaze and participants’ ethnicity on the cone width, we ran a linear mixed model in *R* using the* lme4* package (Bates et al., 2007) with eye gaze (Direct Gaze, Eyes Closed) and participant ethnicity (East Asian, White) as well as their interaction as fixed effects and participant as a random effect. No random slopes were included in the model. Significance was established via log-likelihood tests by dropping effects of interest from the appropriate model: For example, the significance of the two-way interaction can be established by comparing a model including a two-way interaction to a model including only the two main effects. Data are plotted in Fig. 2b.

As predicted, eye gaze had a significant effect on cone width, χ^2^(1) = 16.28, *p* < .001, with the model estimating that the cone was 2.87° (CI [1.1°, 4.64°]) wider in the Direct Gaze condition compared with the Eyes Closed condition. There was neither a main effect of participant ethnicity, χ^2^(1) < 0.01. *p* = .923, nor was there an interaction between eye gaze and participant ethnicity, χ^2^(1) = .027, *p* = .606.

Post hoc tests implemented using *emmeans* (Lenth, 2016) confirmed that the effect of direct gaze on cone width could be observed in both White, *t*(123) = 3.17, *p* = .002, and East Asian, *t*(123) = 2.71, *p* = .008, participant samples.

### Discussion

In line with our predictions, we find that the “ventriloquism effect” is modulated by socially salient eye gaze information, such that perceived voice locations are drawn toward the location of a face that is directly looking at participants. We note that the ventriloquism effect appears to be asymmetrical, being more pronounced for sounds located to the left of the participant. We further found no evidence for an “other-race” effect in our data (e.g., Hancock & Rhodes, 2008; Hugenberg et al., 2010; Rhodes et al., 2009).

While Experiment 1 shows clearly that direct eye gaze attracts perceived sound locations significantly more than a face shown with eyes closed, it is unclear how this effect of eye gaze relates to the “ventriloquism effect” per se, where the perceived location of a sound is drawn toward any visual stimulus.

## Experiment 2

To examine how our reported effect related to the ventriloquism effect and to replicate our findings from Experiment 1, we conducted a follow-up experiment in which we included an audio-only baseline condition. In this baseline condition, participants were only presented with sounds presented in different locations in space, in the absence of a visual stimulus. If our reported effects are indeed tapping into the ventriloquism effect, we should observe that cone widths are narrower for this audio-only baseline conditions compared with both the Direct Gaze and Eyes Closed conditions, as both images of faces should attract perceived sound locations.

### Methods

#### Participants

A total of 100 participants were recruited from the testing platform Prolific. All participants were between 18 and 40 years old, were White, reported no hearing difficulties, had normal or corrected-to-normal vision, and had an approval rate of more than 90% on Prolific. The data from 76 participants is presented in the analysis below (see Data Processing for exclusion criteria). These participants were on average 24.7 years old (*SD* = 5.01 years), and 32 of these participants identified as female.

The study was approved by the local ethics committee at Queen Mary University of London (ethics number: QMERC2498a). All participants were reimbursed for their time (£1.30 for approximately 12 minutes of participation).

#### Materials and procedure

The materials used for this experiment were identical to the ones used for Experiment 1. The procedure for the task was also identical to Experiment 1, with one difference being that 90 baseline trials were added to the experimental task. During these 90 baseline trials participants were presented with the auditory stimuli as in Experiment 1, with no accompanying visual stimulus. There were in total 270 trials in this experiment, plus 12 vigilance trials. The order of trials was fully randomized across participants. A second difference to Experiment 1 was that no other tasks beyond the information and consent form preceded the ventriloquism task.

#### Data processing

Data were processed in the same way as for Experiment 1. Out of the 100 participants tested, 24 were excluded: Three participants failed more than 20% of vigilance trials; four participants were excluded because we were unable to fit required logistic functions to their data; and 17 participants were removed because accuracy for one or more of the easiest conditions (−40° and +40° azimuth; see Materials for Experiment 1) was lower than 80%.

### Results

**Fig. 3:**
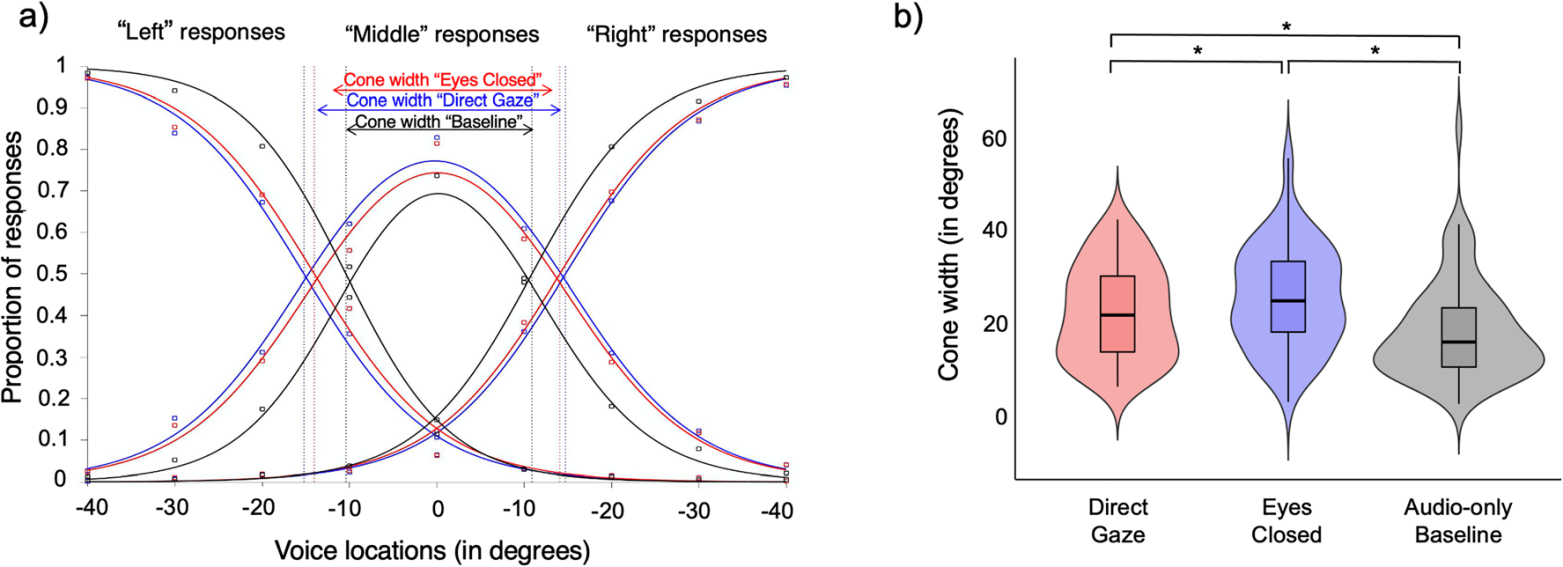
**a** Plot showing the functions fitted for average “left,” “right,” and “middle” responses in the “Eyes Closed” (red), “Direct Gaze” (blue) and “Baseline” (i.e., audio-only; black) conditions. The dashed lines mark the intersections between the functions fitted to the “left” and “right” responses, respectively, and the functions fitted to the “middle” responses per gaze condition. The arrows show the cone width per gaze condition. **b** Violin plots showing the data for the three visual conditions *p < .05. (Colour figure online)

As in Experiment 1, we examined the effects of the visual stimulus on the cone width via a linear mixed model in *R* using the *lme4* package (Bates et al., 2007) with visual stimulus (Face with Direct Gaze, Face with Eyes Closed, Audio-Only Baseline) and participant as a random effect. No random slopes were included in the model. Significance was again established via log-likelihood tests by dropping the fixed effect of visual stimulus from the model of interest. Data are plotted in Fig. 3.

The nature of the visual stimulus had a significant effect on cone width, χ^2^(2) = 35.79, *p* < .001. Post hoc tests implemented using *emmeans* (Lenth, 2016) confirmed that all three types of visual stimuli resulted in significantly different cone widths (Fig. 3b). The model estimated that the cone was 3.22° wider in the Direct Gaze condition compared with the Eyes Closed condition, *t*(154) = 2.77, *p* = .006, replicating our findings from Experiment 1. The model also estimated the cones for the Eyes Closed condition was 4.10° wider than in the Audio-Only Baseline condition, *t*(154) = 3.53, *p* < .001. Finally, the model estimated the cones for the Direct Gaze condition was 7.33° wider than in the Audio-Only Baseline condition, *t*(154) = 6.30, *p* < .001.

### Discussion

In Experiment 2 we replicate our findings from Experiment 1, showing that direct eye gaze enhances the ventriloquism effect. We further directly relate the observed effect to the “classic” ventriloquism effect. By comparing sound location perception in the absence of visual information to perception in the presence of visual information (Direct Gaze and Eyes Closed condition), we showed that participants are overall more likely to perceive sound locations to come from a central position and thus coincide with the location of a visual stimulus than when no visual stimulus is present.

## General discussion

The findings of our experiments can help understand the processes that can give rise illusory percepts through multisensory or in this case audiovisual integration. While it was traditionally thought that the ventriloquism effect relies largely on automatic processes and arises from low-level audiovisual integration, our findings align well with other, generally more recent, reports showing that the ventriloquism effect can—under some circumstances—be modulated by top-down influences (Bruns, 2019; Bruns et al., 2014; Delong & Noppeney, 2021; Driver, 1996; Radeau & Bertelson, 1977; Thurlow & Jack, 1973). Specifically, our experiments add to a literature that shows that although the ventriloquism effect can be readily induced from any combination of audio-visual stimuli, the specific type of visual stimulus matters and can modulate the size of the ventriloquism effect. Across two experiments, we have shown that the ventriloquism effect is enhanced by faces exhibiting direct eye gaze, such that listeners’ perception of sound locations was pulled more toward the location of these faces, compared with faces that were shown with their eyes closed.

Which features of our stimulus manipulation, contrasting a face exhibiting direct gaze with a face with its eyes closed, may have driven the enhancement of the ventriloquism effect? First, it may be that differences in physical image properties between the two conditions could have driven this effect. However, the image properties across conditions were very closely matched with the stimuli showing a face with its eyes closed being derived from the original “direct gaze” images, such that only the eye regions differed between conditions (see Fig. 1b). However, several studies have shown that the size of the ventriloquism effect did not differ when visual stimuli with dramatically different physical properties were used (e.g., comparing the size of the ventriloquism effect elicited by using faces vs. flashes of lights; Radeau & Bertelson, 1977). As such it seems unlikely that the small differences in image properties led to the effect reported in our experiments.

Direct gaze has been described as a self-relevant signal that reorients the viewer’s attention toward the directly gazing face (e.g., Mares et al., 2016). As such, it is possible that the enhanced ventriloquism effect in the presence of direct gaze is driven by the attentional enhancement afforded to the direct gaze versus closed eyes. However, conversely, earlier studies of the ventriloquism effect have shown that the effect is in fact not influenced by visual attention—be it deliberate or automatic (Bertelson et al., 2000; Vroomen et al., 2001). These studies would therefore suggest that our observed effect is unlikely to be attributable to differences in attention. An explanation that may resolve these conflicting findings related to our study may be found in how our study differs from Bertelson et al. (2000) and Vroomen et al. (2001), who report that attention does not modulate the ventriloquism effect. One key difference between these studies and ours is the use of complex and socially-salient audiovisual pairings of faces and voices, while the earlier studies have tested the ventriloquism effect using more low-level auditory and visual stimuli (e.g., bursts of noise and flashes of light). Although stimulus complexity per se does not necessarily significantly affect the size of the ventriloquism effect itself (Radeau & Bertelson, 1977), increasing complexity and ecological validity of stimuli has been argued to facilitate the detection of top-down influences on the ventriloquism effect (e.g., semantic congruency effects; Delong & Noppeney, 2021). 

In the context of this wealth of studies pointing to eye gaze modulating attention (Mares et al., 2016; Senju & Hasegawa, 2005), we argue that our findings may therefore still be related to modulations of attention linked to direct gaze. We propose that certain aspects of the eye gaze—related to the social salience of eyes and/or the self-referential nature of direct gaze and enhanced by the complexity of our stimuli—may have modulated attention of participants, resulting in the observed effect. Since Vroomen et al. (2001) and Bertelson et al. (2000) did not manipulate such complex, socially driven aspects of attention when using low-level stimuli, our results are therefore not necessarily in direct conflict with their findings. An alternative explanation could be found in perspectives that propose that perceivers integrate signals across modalities to minimize stimulus ambiguity. This is thought to be achieved by weighting the information in sensory modalities in relation to their informativeness or reliability (e.g., Alais & Burr, 2004; Ernst & Banks, 2002; Fetsch et al., 2013). In our experiments, the presence of direct eye gaze could turn the visual stimulus into a source of information that is perceived to be more reliable, which could then result in it being afforded greater weight during multisensory integration than the less salient face without direct gaze. If the visual information, that is presented in a central position is weighted more heavily, we would observe that sound locations should be more strongly attracted by the face exhibiting direct eye gaze, thus enhancing of the ventriloquism effect for direct gaze. We, however, acknowledge that these interpretations need to remain speculative as it is difficult to dissociate the specific mechanisms supporting our effect based on the current data.

Finding no evidence for an “other-race” effect was initially surprising in Experiment 1 as some other-race effects have previously been reported in the perception of direct gaze (e.g., Collova et al., 2017; Pavan et al., 2011). The existing literature on other-race effects proposes that  other-race effects may be the result of a combination of reduced perceptual expertise and increased category-based (as opposed to individual-based) processing of other-race faces (Hugenberg et al., 2010). Given that the ventriloquist effect can be observed with a range of audiovisual stimulus pairings, some of them entirely novel to participants, without significant changes in the size of the effect (e.g., Bertelson et al., 1994; Colin et al., 2001; Radeau & Bertelson, 1977), one might anticipate that the degree of perceptual expertise with processing a specific type of stimulus should not impact the ventriloquist effect. Similarly, the ethnicity of the participants was not salient in our experiment, such that not finding an effect indeed aligns with Pavan et al.’s (2011) findings who found that situational salience was a requirement for other-race effect in gaze perception to emerge. As such, upon closer inspection of the literature, not finding an other-race effect appears to indeed align well with previous findings. We note, however, that the lack of an other-race effect in our experiment should overall be treated with some caution, as the analysis was exploratory and only White facial stimuli were included, such that the experimental design was not balanced.

In addition to highlighting the role of top-down influences on the ventriloquism effect and potentially on audiovisual integration more generally, our data also overall suggest that outside of the laboratory, classic ventriloquism performances are fine-tuned to yield a strong and maximally compelling perceptual illusion. To achieve this, performers focus on establishing an interactional style between the puppet and the ventriloquist that serves to enhance the strength of the illusion; this includes coordinated suppression and expression of mouth movements and—as established here—coordinated eye gaze. More broadly, the misattribution or fusion of sound locations with visual information is by no means restricted to ventriloquism performances but is also experienced in everyday life. For example, when attending a lecture where the presenter’s voice is amplified through loudspeakers from a number of locations in the venue, audiences rarely perceive the location of the speaker and the amplified voice as being discrepant.

Similarly, in neuropsychiatric disorders such as schizophrenia, patients are often reported to experience auditory (verbal) hallucinations, where sounds—be they sounds that are misperceived or sound percepts originating from e.g. the patient’s experience of their inner speech—are perceived to be coming from a specific location in space (Plaze et al., 2011; Shergill et al., 2000). With illusory effects relying on multisensory integration, such as the McGurk effect, having been observed to be weaker in patients with schizophrenia (Vanes et al., 2016; White et al., 2014), the mechanisms underpinning such auditory (verbal) hallucinations have indeed been ascribed to a dysfunction of multisensory integration. More work is, however, still needed to better understand how audiovisual integration works and under which circumstances some factors are able to exert top-down modulations on these integration processes both healthy populations as well as populations with, for example, neuropsychiatric disorders. Our study makes a step in this direction, by highlighting direct eye gaze as one such factor.
